# Clinical characteristics and treatment of actinomycetoma in northeast Mexico: A case series

**DOI:** 10.1371/journal.pntd.0008123

**Published:** 2020-02-25

**Authors:** Jesús Alberto Cárdenas-de la Garza, Oliverio Welsh, Adrián Cuéllar-Barboza, Karina Paola Suarez-Sánchez, Estephania De la Cruz-Valadez, Luis Gerardo Cruz-Gómez, Anabel Gallardo-Rocha, Jorge Ocampo-Candiani, Lucio Vera-Cabrera

**Affiliations:** Servicio de Dermatología, Facultad de Medicina y Hospital Universitario “Dr. José Eleuterio González”, Universidad Autónoma de Nuevo León, Monterrey, México; Centers for Disease Control and Prevention, UNITED STATES

## Abstract

**Background:**

Mycetoma is a neglected tropical disease characterized by nodules, scars, abscesses, and fistulae that drain serous or purulent material containing the etiological agent. Mycetoma may be caused by true fungi (eumycetoma) or filamentous aerobic bacteria (actinomycetoma). Mycetoma is more frequent in the so-called mycetoma belt (latitude 15° south and 30° north around the Tropic of Cancer), especially in Sudan, Nigeria, Somalia, India, Mexico, and Venezuela. The introduction of new antibiotics with fewer side effects, broader susceptibility profiles, and different administration routes has made information on actinomycetoma treatment and outcomes necessary. The objective of this report was to provide an update on clinical, therapeutic, and outcome data for patients with actinomycetoma attending a reference center in northeast Mexico.

**Methodology/principal findings:**

This was a retrospective, cross-sectional, descriptive study of 31 patients (male to female ratio 3.4:1) diagnosed with actinomycetoma by direct grain examination, histopathology, culture, or serology from January 2009 to September 2018. Most lesions were caused by *Nocardia brasiliensis* (83.9%) followed by *Actinomadura madurae* (12.9%) and *Actinomadura pelletieri* (3.2%). About 50% of patients had bone involvement, and the right leg was the most commonly affected region in 38.7% of cases. Farmers/agriculture workers were most commonly affected, representing 41.9% of patients. The most commonly used treatment regimen was the Welsh regimen (35.5% of cases), a combination of trimethoprim/sulfamethoxazole (TMP/SMX) plus amikacin, which had a 90% cure rate, followed by TMP/SMX plus amoxicillin/clavulanic acid in 19.4% of cases with a cure rate of 100%. In our setting, 28 (90.3%) patients were completely cured and three (9.7%) were lost to follow-up. Four patients required multiple antibiotic regimens due to recurrences and adverse effects.

**Conclusions/significance:**

In our sample, actinomycetoma was predominantly caused by *N*. *brasiliensis*. Most cases responded well to therapy with a combination of TMP/SMX with amikacin or TMP/SMX and amoxicillin/clavulanic acid. Four patients required multiple antibiotics and intrahospital care.

## Author summary

Mycetoma is a chronic skin disease that can invade bone or underlying organs. Inadequate treatment may lead to long-lasting disability. Information about treatment is scarce. We report the outcomes of 31 patients with a diagnosis of bacterial actinomycetoma attending a tertiary care hospital in northeast Mexico. Most cases were treated with a combination of antibiotics including TMP/SMX plus amikacin or TMP/SMX plus amoxicillin/clavulanic acid. Cure was achieved in 90% of patients and only one required surgery.

## Introduction

Mycetoma is a chronic subcutaneous granulomatous infection characterized by firm swellings and the presence of nodules, scars, abscesses, and fistulae that drain serous or purulent material containing the etiological agent. Mycetoma can be caused by true fungi (eumycetoma) or filamentous aerobic bacteria (actinomycetoma) [1–3]. Most mycetoma cases are concentrated in the so-called mycetoma belt located between 15° latitude south and 30° latitude north around the Tropic of Cancer. Eumycetoma cases mainly occur in Sudan, Senegal, Nigeria, Somalia, Mauritania, and India [2], while most actinomycetoma cases have been reported in Mexico and Venezuela, although sporadic cases have also been reported in the United States and Europe. Endemic regions have a predominantly subtropical and tropical dry climate with low humidity and an annual rainfall between 50 and 1000 mm [1–3]. Specific etiological agents vary accordingly to climate and geography, with actinomycetomas predominating in drier regions. The most frequently affected sites are the lower limbs followed by the arms and trunk [4]. Actinomycetoma is usually more aggressive than eumycetoma, spreading more quickly and with a greater tendency to be extrapedal than eumycetoma [5].

Mycetoma therapy differs depending on its etiology. Eumycetoma treatment primarily relies on wide surgical excision of the lesion and administration of antifungal agents, while actinomycetoma therapy is based on antimicrobials. Sulfonamides, particularly trimethoprim-sulfamethoxazole (TMP/SMX), have hitherto been the mainstay therapy, often in combination with other drugs such as amikacin, amoxicillin/clavulanic acid, rifampicin, or DDS (diamino-diphenyl-sulfone) [6]. However, treatment guidance is scarce and mostly based on case reports or small case series of less than 20 cases [5, 6]. Retrospective epidemiological reports often do not include treatment and outcome details. Although cure can be achieved with adequate antibiotic therapy, the limited available evidence makes actinomycetoma treatment a challenge for many physicians.

The World Health Organization (WHO) included mycetoma as a neglected tropical disease in 2016 [7], which has encouraged research into the topic and identified several knowledge gaps. Epidemiological changes due to urbanization and migration have influenced a shift in mycetoma patient demographics. Furthermore, the introduction of new drugs with fewer side effects, broader susceptibility profiles, and different administration routes over the last few decades has made information about current actinomycetoma treatment regimens and outcomes necessary. The objective of this report is to provide an update on the clinical, therapeutic, and outcome data for patients with actinomycetoma attending a reference center in northeast Mexico.

## Methods

This was a single-center, ambispective, longitudinal, descriptive study. The protocol was carried out in “Dr. José Eleuterio González” University Hospital in Monterrey, a reference center for mycetoma diagnosis and treatment in northeast Mexico. All patients attending the clinic between January 2009 and September 2018 were included in the analysis.

Diagnosis was made by direct grain examination, histopathology, culture, or serology. Isolates were identified to species level using nucleotide sequence analysis of a fragment of the small ribosomal subunit (16S) gene. Clinical data were retrieved from the clinical files and the image library of the Department of Dermatology. Microbiological data were obtained from the Interdisciplinary Dermatology Research Laboratory. All subjects underwent skin biopsy for histopathological analysis and fungal culture. Patients with exudates underwent direct examination with potassium hydroxide (KOH) and culture. Pathology specimens were examined with hematoxylin and eosin (H&E) and antifungal stains (periodic acid-Schiff and/or Grocott's methenamine silver). Sabouraud dextrose and Mycosel agar (Becton Dickinson, Franklin Lakes, NJ, USA) were used for culture. Anti-*Nocardia brasiliensis* antibodies were assessed using the methodology described by Salinas-Carmona et al. [8]. Patients with negative diagnostic tests by direct examination with KOH, culture, or serology were excluded from the analysis, as were eumycetoma cases.

Treatment was determined according to lesion size, location, bone/internal organ involvement, recurrence, adverse events, comorbidities, and response to previous treatments. TMP/SMX monotherapy was used in patients with lesions under 5 cm. TMP/SMX combined with amoxicillin/clavulanic acid was used in larger lesions without bone/internal organ involvement. TMP/SMX in combination with amikacin was used in recalcitrant lesions involving more than two body regions, internal organ/bone involvement, and special locations (head and neck). Cases resistant to combined TMP/SMX and amoxicillin/clavulanic acid received a triple combination with amikacin. Other cases with adverse events to previous treatment(s), drug allergies, recurrences, or extensive abdominal organ involvement received combination regimens that included moxifloxacin, carbapenems, fosfomycin, or rifampicin, depending on inpatient or outpatient management, availability, comorbidities, and affordability. Cure was defined as remission of the lesions, lack of inflammation, and absence of exudate, as well as negative follow-up skin biopsies.

Statistical analyses were performed using IBM SPSS v.24 (IBM Inc., Armonk, NY, USA). Frequencies and percentages were used to describe categorical variables. Normality was evaluated with the Shapiro-Wilk test. Normally distributed variables were described with means and standard deviations, while non-normally distributed variables were described with medians and interquartile ranges (IQR).

### Ethics statement

The protocol was approved by the institutional ethics and research committee (reference number DE17-00009). Informed written consent was obtained for clinical iconographies and biopsies.

## Results

Thirty-one patients with a diagnosis of actinomycetoma were analyzed, 24 men (77.4%) and seven women (22.6%; ratio 3.4:1) aged between 15 and 73 years of age (median 50, IQR: 22). The median lag time between symptom onset and evaluation at our center was 103.8 months (IQR: 144). The sociodemographic characteristics of the study population are shown in **Table 1**.

**Table 1:** Sociodemographic characteristics.

Characteristics	n (%)
**Gender**	
Male	24 (77.4)
Female	7 (22.6)
**Age**	
11–20	3 (9.7)
21–30	1 (3.2)
31–40	4 (12.9)
41–50	8 (25.8)
51–60	8 (25.8)
61–70	6 (19.4)
>70	1 (3.2)
**Origin**	
Nuevo León	15 (48.4)
San Luis Potosí	8 (25.8)
Tamaulipas	5 (16.1)
Coahuila	2 (6.5)
Veracruz	1 (3.2)
**Occupation**	
Farmer/agricultural worker	13 (41.9)
Construction worker	6 (19.4)
Housewife	4 (12.9)
Gardener	1 (3.2)
Other	7 (22.6)
**History of trauma**	
Reported	12 (38.7)
Denied	10 (32.3)
Did not remember	6 (19.4)
Unknown	3 (9.7)
**Time between symptom onset and evaluation**	
0–12 months	4 (12.9)
13–24 months	3 (9.7)
25–36 months	0 (0)
37–48 months	2 (6.5)
49–61 months	0 (0)
>61 months	21 (67.7)
Unknown	1 (3.2)
**Affected region**	
Trunk	10 (32.3)
Right leg	12 (38.7)
Left leg	6 (19.4)
Both legs	1 (3.2)
Right arm	2 (6.5)
**Bone invasion**	
Positive	15 (48.4)
Negative	16 (51.6)

Most subjects (13/31; 41.9%) were farmers/agricultural workers followed by construction workers (6/31; 19.4%) and housewives (4/31; 12.9%). Fifteen (48.4%) patients lived in the state of Nuevo Leon, eight (25.8%) in San Luis Potosi, and five (16.1%) in Tamaulipas. The right leg was the most commonly affected site in 12 (38.7%) patients, followed by the trunk in ten (32.3%) and the left leg in six (19.4%). A history of trauma was reported in 12 (38.7%) patients. Bone involvement documented by imaging methods was present in 15 (48.4%) cases.

The diagnostic information is presented in **Table 2**. Grain detection by direct examination with KOH was positive in 22 (71%) individuals. Two subjects had small reniform yellow grains suggestive of *Nocardia* spp., and one had large white-yellowish grains suggestive of *A*. *madurae*. Culture of the skin biopsy or swab grew *N*. *brasiliensis* in 15 (48.4%) patients, *Actinomadura madurae* in four (12.9%) patients, and *A*. *pelletieri* in one (3.2%) patient. All culture isolates were identified to species level using nucleotide sequence analysis of a fragment of the small ribosomal subunit (16S) gene. Anti-*N*. *brasiliensis* antibodies were positive in 20 (64.5%) patients. Histopathological analysis demonstrated grains in 14 (45.2%) cases.

**Table 2:** Diagnosis information.

Number	Direct mycological examination	Culture	Biopsy	Anti-*Nocardia* antibodies	Agent
1	Positive	Positive	Chronic granulomatous inflammation	Negative	*N*. *brasiliensis*
2	Negative	Negative	Chronic granulomatous inflammation	Positive	*N*. *brasiliensis*
3	Negative	Negative	Chronic granulomatous inflammation	Positive	*N*. *brasiliensis*
4	Positive	Positive	Compatible^a^	Positive	*N*. *brasiliensis*
5	Positive	Negative	Compatible^a^	Positive	*N*. *brasiliensis*
6	Positive	Positive	Acute and chronic granulomatous inflammation	-	*N*. *brasiliensis*
7	Positive	Positive	Compatible^a^	Negative	*N*. *brasiliensis*
8	Positive	Negative	Chronic granulomatous inflammation	-	^b^*Grains suggestive of A*. *madurae*
9	Positive	Negative	Compatible^a^	Positive	*N*. *brasiliensis*
10	Positive	Positive	Compatible^a^	Positive	*N*. *brasiliensis*
11	Negative	Positive	Acute and chronic granulomatous inflammation	Positive	*N*. *brasiliensis*
12	Positive	Positive	Compatible^a^	Positive	*A*. *madurae*
13	Positive	Positive	Compatible^a^	Positive	*A*. *pelletieri*
14	Positive	Positive	Acute and chronic granulomatous inflammation	Positive	*N*. *brasiliensis*
15	Positive	Positive	Chronic granulomatous inflammation	Negative	*A*. *madurae*
16	Positive	Positive	Compatible^a^	Positive	*N*. *brasiliensis*
17	Positive	Positive	Compatible^a^	Positive	*N*. *brasiliensis*
18	Negative	Positive	Compatible^a^	Positive	*N*. *brasiliensis*
19	Positive	Positive	Chronic granulomatous inflammation	Positive	*N*. *brasiliensis*
20	Positive	Negative	Compatible^a^	Negative	^c^Grains suggestive of *Nocardia* spp.
21	Positive	Positive	Acute and chronic granulomatous inflammation	-	*N*. *brasiliensis*
22	Positive	Positive	Chronic granulomatous inflammation	Negative	*N*. *brasiliensis*
23	Positive	Positive	Chronic granulomatous inflammation	Positive	*A*. *madurae*
24	Positive	Positive	Acute and chronic granulomatous inflammation	Negative	*N*. *brasiliensis*
25	Positive	Negative	Acute and chronic granulomatous inflammation	-	^b^Grains suggestive of *Nocardia* spp.
26	Negative	Negative	Chronic granulomatous inflammation	Positive	*N*. *brasiliensis*
27	Negative	Negative	Acute and chronic granulomatous inflammation	Positive	*N*. *brasiliensis*
28	Negative	Negative	Chronic granulomatous inflammation	Positive	*N*. *brasiliensis*
29	Positive	Positive	Compatible^a^	Negative	*N*. *brasiliensis*
30	Negative	Positive	Compatible^a^	Positive	*A*. *madurae*
31	Negative	Negative	Compatible ^a^	Positive	*N*. *brasiliensis*

^a^Compatible biopsy: presence of grains on histopathological examination

^b^Grains suggestive of *A*. *madurae*: large white-yellowish grains

^c^Grains suggestive of *Nocardia* spp.: reniform, small, and yellow grains.

**Table 3** outlines the treatments received. Twenty subjects had previously received antibiotic treatment, prescribed at other institutions, without improvement. Most of them had received TMP/SMX at unspecified doses and sometimes with different combinations before evaluation at our institution.

**Table 3:** Treatments and outcomes.

Treatment	Patients (%)	Mean duration	Cure	In current treatment	Without follow-up	Months without recurrence, median in months (IQR)^b^
TMP/SMX^a^	4 (12.9)	3.75 months	3	0	1	26.8 (44.25)
TMP/SMX^a^ + amikacin	11 (35.5)	7 months	10	0	1	12.9 (12)
TMP/SMX^a^ + amoxicillin/clavulanic acid	6 (19.4)	8.3 months	6	0	0	7.3 (14.5)
TMP/SMX^a^ + amoxicillin/clavulanic acid + amikacin	4 (12.9)	7 months	3	0	1	6 (18)
TMP/SMX^a^ + amikacin + moxifloxacin	2 (6.5)	15 months	2	0	0	55
Recalcitrant cases	4 (12.9)	22.8 months	4	0	0	26.8 (63.75)

^a^TMP/SMX, trimethoprim/sulfamethoxazole

^b^IQR, interquartile range.

**Fig 1:**
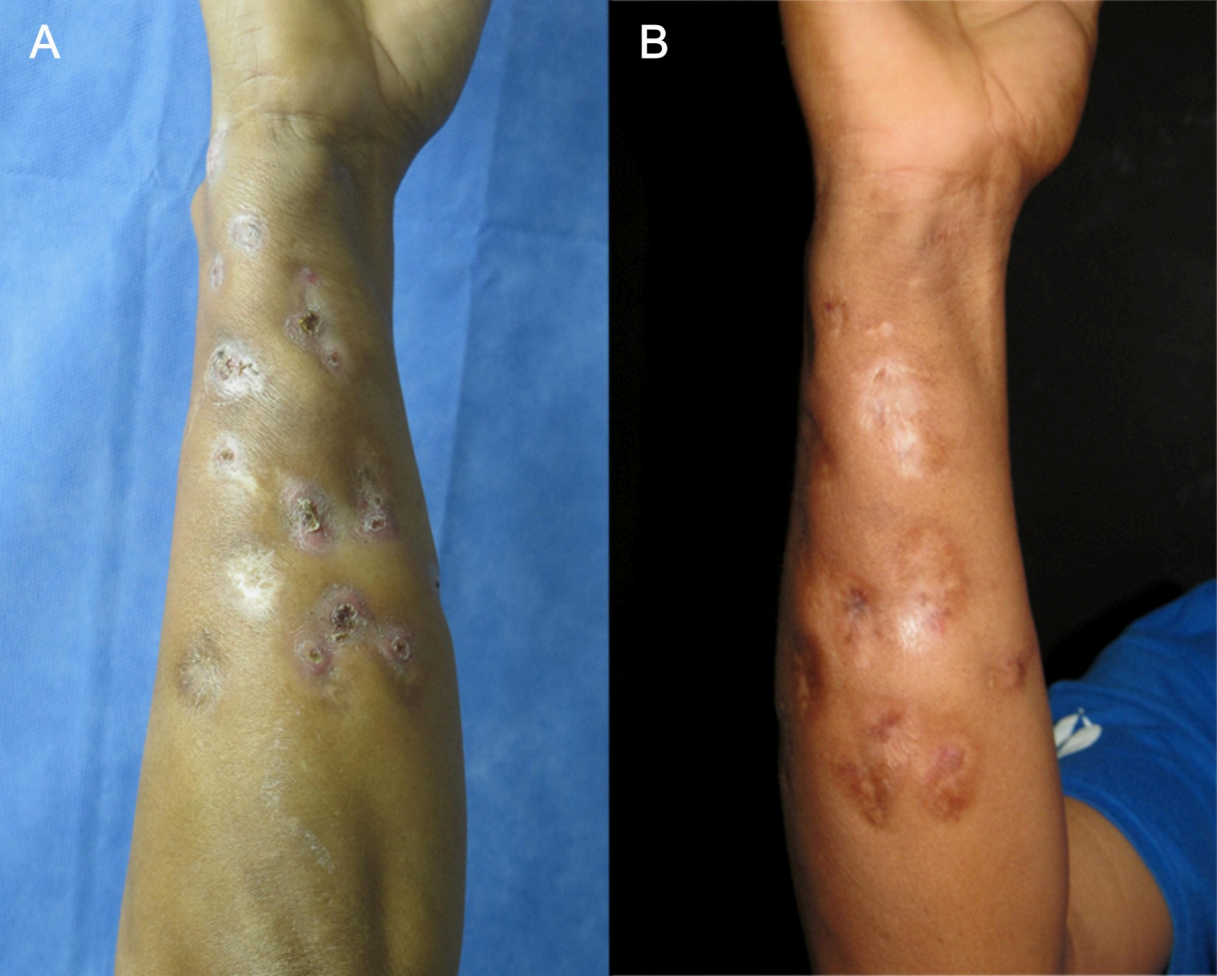
Actinomycetoma treated with trimethoprim/sulfamethoxazole and amikacin. (A) Fourteen-year-old *Nocardia brasiliensis* actinomycetoma on the forearm. (B) Complete resolution after three cycles of trimethoprim/sulfamethoxazole and amikacin.

The most common treatment was the Welsh regimen, a combination of TMP/SMX + amikacin, administered in cycles. Each cycle consisted of amikacin 15 mg/kg/day intramuscularly divided into two daily doses for three weeks simultaneously with TMP/SMX 8/40 mg/kg/day orally for five weeks (**Fig 1**).

This regimen was employed in 11 (35.5%) subjects with previously no response to therapy, bone/internal organ involvement, or lesions located on the thorax, neck, or head.

**Fig 2:**
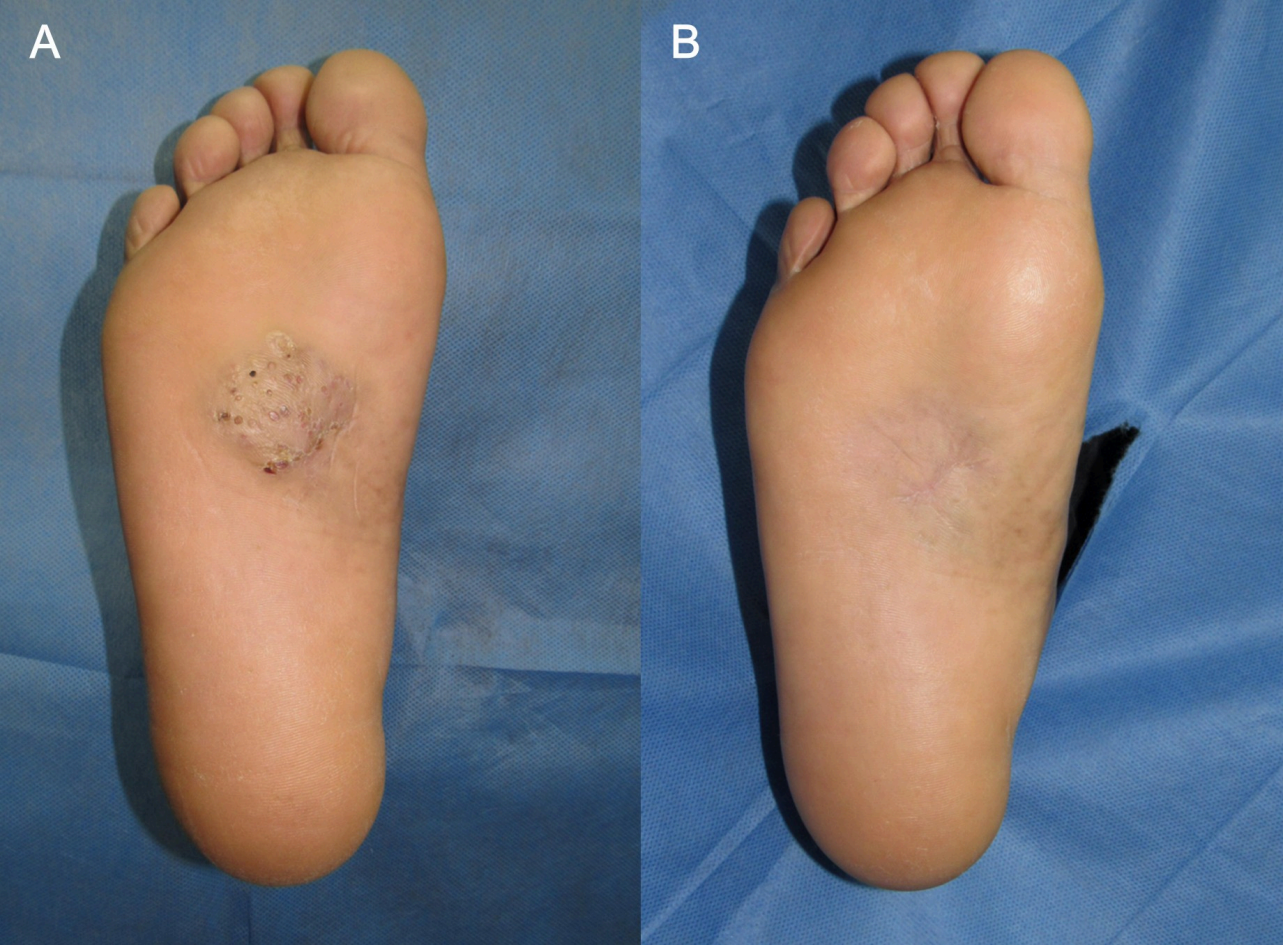
Actinomycetoma treated with trimethoprim/sulfamethoxazole and amoxicillin/clavulanic acid. (A) Two-year-old *Nocardia brasiliensis* mycetoma on the right sole. (B) Complete resolution after 7 months of treatment with trimethoprim/sulfamethoxazole and amoxicillin/clavulanic acid.

The second most frequent combination was TMP/SMX with amoxicillin/clavulanic acid 875/150 mg every 12 hours in six (19.4%) cases (**Fig 2**).

**Fig 3:**
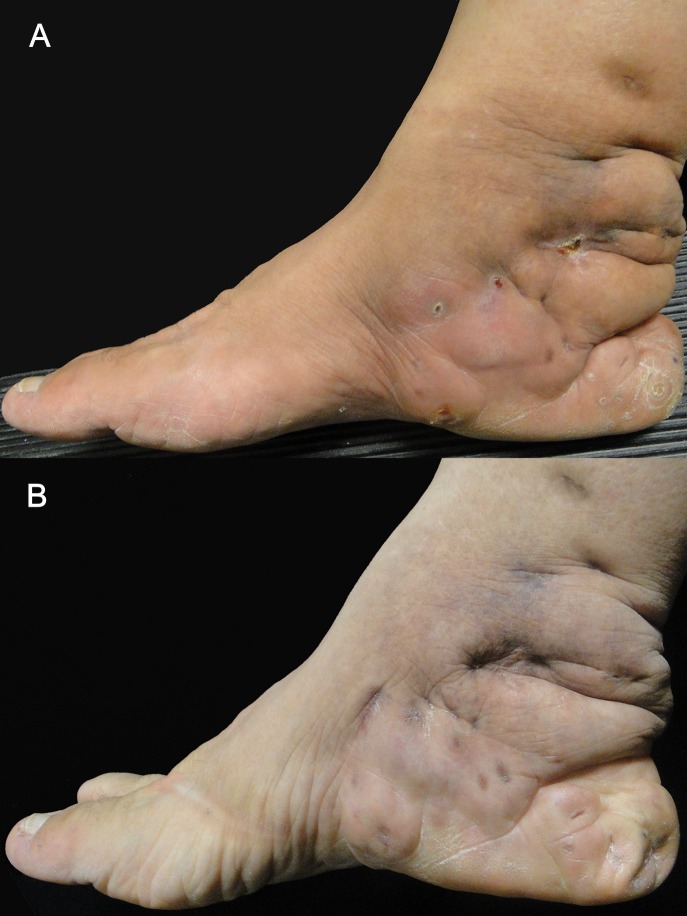
Actinomycetoma treated with trimethoprim/sulfamethoxazole, amikacin, and moxifloxacin. (A) Ten-year-old *Actinomadura madurae* actinomycetoma on the right foot. (B) Complete resolution after 14-months of treatment with trimethoprim/sulfamethoxazole, amikacin, and moxifloxacin.

TMP/SMX therapy was used in four (12.9%) patients. The combination of TMP/SMX, amoxicillin/clavulanic acid, and amikacin was used in four (12.9%) subjects. These patients had previously responded poorly to combined TMP/SMX + amoxicillin/clavulanic acid in our clinic. Two patients that responded poorly or recurred after combined TMP/SMX and amikacin received additional moxifloxacin, resulting in complete cure of the lesion (**Fig 3**).

Four difficult cases due to adverse drug events, recurrence, or extensive abdominal organ involvement required multiple treatment regimens as described in **Table 4**.

**Table 4:** Recalcitrant cases.

Clinical presentation	Causative agent	Previous treatment	Outcome with previous therapy	Successful treatment
54-year-old woman with right thigh and leg involvement	*N*. *brasiliensis*	TMP/SMX^a^ Amoxicillin/ clavulanic acid Minocycline Doxycycline Imipenem Netilmicin Gentamicin	Drug allergy to TMP/SMX^a^, amoxicillin/ clavulanic acid, and moxifloxacin Two relapses and therapeutic failure	Meropenem, amikacin, and rifampicin
51-year-old man with abdominal and pelvic involvement	*N*. *brasiliensis*	None	Extensive abdominal organ involvement	Imipenem, TMP/SMX^a^, amikacin, and moxifloxacin
50-year-old man with right leg and genital area involvement	*N*. *brasiliensis*	TMP/SMX^a^ Amoxicillin/ clavulanic acid Amikacin Streptomycin Dapsone Imipenem	Multiple recurrences Acute kidney injury Extensive bone invasion	Hip disarticulation followed by meropenem, fosfomycin, rifampicin, and TMP/SMX^a^
34-year-old man with involvement of left thigh and bone involvement	*N*. *brasiliensis*	TMP/SMX^a^ Amikacin	Hypoacusia Acute kidney injury	Imipenem, TMP/SMX^a^, and amoxicillin/clavulanic acid

^a^TMP/SMX, trimethoprim/sulfamethoxazole.

Total treatment duration ranged from 1 to 57 months (median 10, IQR: 7); however, the duration was adapted according to disease evolution in each patient. Twenty-eight (90.3%) patients had complete resolution of the infection. The median follow-up after cure was 17.2 months (IQR: 24) with a range of 1–83 months. Three patients (9.7%) were lost to follow-up. Of the four patients treated with TMP/SMX monotherapy, three (75%) had complete cure. Of the 11 patients treated with TMP/SMX + amikacin, ten (90.9%) had complete resolution of the infection. The six patients treated with TMP/SMX + amoxicillin/clavulanic acid were cured. Three (75%) of the four patients treated with TMP/SMX + amikacin + amoxicillin/clavulanic acid had complete resolution.

Amikacin adverse events were detected in six patients. This antibiotic had to be suspended in two patients, in one due to acute kidney injury and in the second due to acute kidney injury and hypoacusia. Reversible creatinine elevation and hypoacusia that did not warrant discontinuation were reported in one (3.2%) and three (9.7%) cases, respectively.

## Discussion

Here we analyzed the clinical and therapeutic characteristics of 31 patients with actinomycetoma predominantly caused by *N*. *brasiliensis*. The male to female ratio in our series was 3.4:1, similar to other Mexican series reporting predominantly actinomycetoma and a male to female ratio of 3.1 and 2.8 to 1 [2, 4]. A review of 21 cases in Rio de Janeiro, Brazil had a male to female ratio of 1.3:1 [7], while data from Africa on predominantly eumycetomas reported a ratio male to female ratio of 3.9, 3.1, and 1.5 to 1 in Senegal, Sudan, and Togo, respectively [9–11]. Only data from Thailand and Tunisia have reported a female predominance [12]. Several factors influence this gender bias including "outdoor" work activity that varies depending on the country and reporting bias. Estrogens may protect against this disease, while progesterone and testosterone may be proinflammatory [3]. This relationship is also influenced by the level of care of the centers surveyed [1].

The most common age range of affected individuals was 41 to 60 years. This differs from other large registries that reported 20 to 40-year-olds as the most commonly affected age group [2, 10]. The patients’ professions were relatively constant between 1992 and 2013 in reports from Mexico, with farmers/agricultural workers and housewives representing close to 60% and 20% of all cases, respectively [4, 13]. Our sample reflects a reduction in affected agricultural workers to 41.9% and an increase in construction workers to 19.4%, which may reflect urbanization of northeast Mexico.

Almost 30% of cases reported here had trunk involvement. The regional difference reported for trunk involvement in the Americas compared to Africa, where the prevalence of trunk involvement is less than 2%, may be partially explained by the regional custom of carrying wood, plant products, and soil on the back [12]. In our series, most patients with back involvement routinely reported transporting agricultural products and wood logs in this way.

In our patients, bone involvement including periostitis, bone erosions, or cavitations was documented by imaging studies in 48.4% of patients, similar to the prevalence encountered in Brazil [7] but much lower than in Togo and Sudan (17–18%) [10, 11]. Many factors influence bone invasion including the osteophilic affinity of the microorganism, the host immune status, and evolution time [14]. Imaging methods, especially contrast-enhanced magnetic resonance imaging, help in the differential diagnosis and in determining optimal therapy. Extensive osteolytic damage helps to determine the most appropriate treatment scheme and prognosis [6]. Imaging availability in some resource-limited endemic regions may limit this evaluation and influence reports of bone involvement in some of the literature.

In our case series, grains were detected more frequently by direct examination (71%) than by biopsy (45.2%). Culture was positive in 64.5% of cases. Ideally, patients with a suspicion of actinomycetoma should undergo direct mycological examination to detect the presence of grains and skin biopsy for culture and histopathological examination. PCR and anti-*Nocardia* antibodies are useful in recalcitrant, unresponsive cases or when the diagnosis is uncertain. The sensitivity and specificity of mycetoma diagnosis assays are highly variable depending on the common etiological agents in the area, the methodology employed, and the experience of the interpreter. ELISA serology as described by Salinas-Carmona et al. [8] has been employed as an aid to the diagnosis and monitoring of treatment responses in *N*. *brasiliensis* mycetoma patients, but no study has assessed the ability of this method to distinguish between *Nocardia* mycetomas or other nocardial infections. While serology alone is not diagnostic of actinomycetoma, the diagnosis can be established by the combination of the clinical presentation (subcutaneous mass, sinuses, and discharge) and positive serology in the setting of an endemic geographic region. Other nocardial infections have a completely different dermatological clinical presentation, which helps in the differential diagnosis of these entities. Due to the rarity of this infection, the sensitivity and specificity of this assay for mycetoma diagnosis have not been evaluated.

In our series, four cases with *Actinomadura* spp. mycetomas had positive serology for *N*. *brasiliensis*. Whether this is secondary to cross-reactivity between the anti-26 and anti-24 kDa protein antibodies in the assay and different antigens in these species has yet to be determined. A combination of techniques depending on resource availability is recommended. When accessible, referral of patients or samples to specialized centers is encouraged.

Controlled clinical studies on actinomycetoma treatment are lacking, and antibiotic regimens are based primarily on case series [1]. Treatment evidence originates mainly from highly endemic countries such as Sudan, Senegal, Mexico, and India [6]. After success with dapsone or streptomycin monotherapy in the late 1960s, TMP/SMX became the gold standard treatment for actinomycetoma, although monotherapy showed variable cure rates [15]. Since then, studies have shown that mainstream therapy must include a combination of antimicrobials rather than relying on a single drug to avoid resistance. Nevertheless, across endemic countries, there is no consensus on the optimal treatment. While often recommended in first-line regimens, evidence on *in vitro* dapsone activity against actinomycetes is scarce. However, there are data on *in vitro* activity against *N*. *brasiliensis*; in a study evaluating the *in vitro* activity of DDS against 30 *N*. *brasiliensis* strains, 90% of isolates were inhibited at a concentration of 32 μg/mL. Due to the lack of cutoff points to determine DDS susceptibility and resistance, further studies correlating this finding with *in vivo* activity are needed [16].

Highlighting the need for more actinomycetoma treatment and prognosis information, a systematic review of reports of mycetoma treatment between 1950 and 2016 revealed only 21 actinomycetoma cases with assessable outcomes (cure, recurrence, or mortality) [17]. Nevertheless, the systematic review did not include important Sudanese, Indian, Senegalese, and Mexican case series previously published and synthesized by Zijilstra et al. in 2016 [5].

Patients refractory or resistant to previous treatment and/or with involvement of underlying organs or bone improved substantially with the combination of amikacin and TMP/SMX. We previously reported fifty-six actinomycetoma patients treated this way in our center, with a >90% cure rate. This regimen is given in five-week cycles, and renal and audiometric monitoring is recommended after each cycle [5, 15, 18]. In the current series, we increased the successful experience of this therapeutic regimen and highlight the need for renal and audiometric monitoring. In settings where this is not possible, other combinations are encouraged.

Most *in vitro* studies of *N*. *brasiliensis* isolates have reported amoxicillin/clavulanate susceptibility [19, 20]. Successful treatment of *Nocardia* actinomycetomas with amoxicillin/clavulanate as rescue therapy after failure with amikacin, TMP/SMX, or dapsone was reported in 19 of 21 patients [21]. This combination was employed in six patients in our series who had lesions ≥5 cm without bone/internal organ involvement and with good tolerance and compliance. This combination does not need periodic laboratory monitoring. If nephrotoxicity or ototoxicity occur during amikacin administration or if audiometry and periodic creatinine clearance evaluation are unavailable, amoxicillin/clavulanate with TMP/SMX represents a good therapeutic alternative in *N*. *brasiliensis* actinomycetoma. *A*. *madurae* has shown *in vitro* resistance to amoxicillin/clavulanate, so other therapeutic schemes with sulphonamides and aminoglycosides are recommended [22, 23]. In countries with a high prevalence of *A*. *madurae* actinomycetomas, other initial combinations should be considered before species confirmation.

A triple combination with amikacin was employed when patients did not respond to previous amoxicillin/clavulanate + TMP/SMX. One subject was lost to follow-up and three were cured. Although there are reports of the use of this regimen, information on its efficacy is scarce [22].

Imipenem and meropenem have demonstrated clinical efficacy for *Nocardia*, *Actimadura*, and *Streptomyces* actinomycetomas, but evidence is still limited to few cases [24–26]. Our case series expands the published clinical experience with combinations including imipenem or meropenem, with three challenging cases resolving after multiple relapses and adverse events to other drugs. These antibiotics have the advantage of having fewer adverse events compared to aminoglycosides. Judicious use of carbapenems is warranted due to growing antibiotic resistance. Data on cases resistant to multiple treatment regimens, experiencing serious drug adverse events, or with internal organ involvement are limited. When considering treatment options in these cases, factors that must be considered include availability, affordability, inpatient or outpatient management, comorbidities, and patient preference. A multidisciplinary team with dermatologists, infectious disease specialists, and mycologists may be necessary.

Limitations of this report include its retrospective, single-center design, the multiple treatment regimens employed, and the total number of cases. Furthermore, all the cases came from a tertiary referral center and may not accurately represent the characteristics of actinomycetoma cases seen in primary or secondary care. Strengths include the detailed diagnostic methods, the molecular species confirmation in over half of the cases, and the follow-up information.

Clinical and therapeutic information on this neglected tropical disease is needed to elaborate public health strategies to reduce disease burden. Collecting regional data helps refine diagnostic and treatment protocols for case management [27]. Ideally, controlled clinical trials need to be performed to determine the best treatment for actinomycetoma, but the low prevalence and the variety of etiological agents limit the ability to conduct these trials. Our case series expands current knowledge on the clinical responses of actinomycetomas to therapy in the Americas. More case series with larger sample sizes and prospective follow-up are necessary to determine the best therapeutic protocol. Environmental studies to determine primary reservoirs of the causative bacteria and case mapping may additionally help in the development of effective preventive strategies and focus efforts on high-burden areas.

In conclusion, here we report 31 actinomycetoma cases treated in a reference center in northeast Mexico. The most common etiological agent was *N*. *brasiliensis* followed by *A*. *madurae* and *A*. *pelletieri*. Almost 50% had bone involvement. Most cases responded to a two or three drug combination involving TMP/SMX, amikacin, and amoxicillin/clavulanate. Three challenging cases had multiple drug adverse effects and recurrences that required multiple treatment schemes to achieve cure.

## References

[1] Nenoff P, Van De Sande WWJ, Fahal AH, Reinel D, Schöfer H. Eumycetoma and actinomycetoma—An update on causative agents, epidemiology, pathogenesis, diagnostics and therapy. J Eur Acad Dermatol Venereol. 2015;29(10):1873–83. 10.1111/jdv.13008 25726758

[2] Bonifaz A, Tirado-Sanchez A, Calderon L, Saul A, Araiza J, Hernandez M, et al Mycetoma: experience of 482 cases in a single center in Mexico. PLoS Negl Trop Dis. 2014;8(8):e3102 10.1371/journal.pntd.0003102 25144462 PMC4140667

[3] Vera-Cabrera L, Salinas-Carmona MC, Waksman N, Messeguer-Perez J, Ocampo-Candiani J, Welsh O. Host defenses in subcutaneous mycoses. Clin Dermatol. 2012;30(4):382–8. 10.1016/j.clindermatol.2011.09.008 22682185

[4] Lopez-Martinez R, Mendez-Tovar LJ, Bonifaz A, Arenas R, Mayorga J, Welsh O, et al [Update on the epidemiology of mycetoma in Mexico. A review of 3933 cases]. Gac Med Mex. 2013;149(5):586–92. 24108347

[5] Zijlstra EE, van de Sande WWJ, Welsh O, Mahgoub ES, Goodfellow M, Fahal AH. Mycetoma: a unique neglected tropical disease. The Lancet Infectious Diseases. 2016;16(1):100–12. 10.1016/S1473-3099(15)00359-X 26738840

[6] Welsh O, Al-Abdely HM, Salinas-Carmona MC, Fahal AH. Mycetoma medical therapy. PLoS Negl Trop Dis. 2014;8(10):e3218 10.1371/journal.pntd.0003218 25330342 PMC4199551

[7] Sampaio FM, Wanke B, Freitas DF, Coelho JM, Galhardo MC, Lyra MR, et al Review of 21 cases of mycetoma from 1991 to 2014 in Rio de Janeiro, Brazil. PLoS Negl Trop Dis. 2017;11(2):e0005301 10.1371/journal.pntd.0005301 28192433 PMC5336304

[8] Salinas-Carmona MC, Welsh O, Casillas SM. Enzyme-linked immunosorbent assay for serological diagnosis of Nocardia brasiliensis and clinical correlation with mycetoma infections. J Clin Microbiol. 1993;31(11):2901–6. 8263174 10.1128/jcm.31.11.2901-2906.1993 PMC266152

[9] Ndiaye D, Ndiaye M, Sene PD, Diouf MN, Diallo M, Faye B, et al [Mycetomas diagnosed in Senegal from 2008 to 2010]. J Mycol Med. 2011;21(3):173–81. 10.1016/j.mycmed.2011.07.003 24451559

[10] Fahal A, Mahgoub el S, El Hassan AM, Abdel-Rahman ME. Mycetoma in the Sudan: an update from the Mycetoma Research Centre, University of Khartoum, Sudan. PLoS Negl Trop Dis. 2015;9(3):e0003679 10.1371/journal.pntd.0003679 25816316 PMC4376889

[11] Darré T, Saka B, Mouhari-Toure A, Tchaou M, Dorkenoo AM, Doh K, et al Mycetoma in the Togolese: An Update from a Single-Center Experience. Mycopathologia. 2018;183(6):961–5. 10.1007/s11046-018-0260-y 29557534 PMC6305724

[12] van de Sande WW. Global burden of human mycetoma: a systematic review and meta-analysis. PLoS Negl Trop Dis. 2013;7(11):e2550 10.1371/journal.pntd.0002550 24244780 PMC3820768

[13] Lopez Martinez R, Mendez Tovar LJ, Lavalle P, Welsh O, Saul A, Macotela Ruiz E. [Epidemiology of mycetoma in Mexico: study of 2105 cases]. Gac Med Mex. 1992;128(4):477–81. 1308000

[14] Arenas R, Fernandez Martinez RF, Torres-Guerrero E, Garcia C. Actinomycetoma: an update on diagnosis and treatment. Cutis. 2017;99(2):E11–e5. 28319638

[15] Welsh O, Vera-Cabrera L, Welsh E, Salinas MC. Actinomycetoma and advances in its treatment. Clin Dermatol. 2012;30(4):372–81. 10.1016/j.clindermatol.2011.06.027 22682184

[16] Gonzalez-Benavides N, Vera-Cabrera L, Sanchez-Meza E, Ocampo-Candiani J, Welsh O. Diaminodiphenyl-sulphone: in vitro activity alone and in combination with other antimicrobials against 30 strains of N. brasiliensis. J Eur Acad Dermatol Venereol. 2019 10.1111/jdv.15660 31063602

[17] Salim AO, Mwita CC, Gwer S. Treatment of Madura foot: a systematic review. JBI Database System Rev Implement Rep. 2018;16(7):1519–36. 10.11124/JBISRIR-2017-003433 29995713

[18] Welsh O, Sauceda E, Gonzalez J, Ocampo J. Amikacin alone and in combination with trimethoprim-sulfamethoxazole in the treatment of actinomycotic mycetoma. J Am Acad Dermatol. 1987;17(3):443–8. 10.1016/s0190-9622(87)70227-8 3308980

[19] Brown-Elliott BA, Brown JM, Conville PS, Wallace RJ Jr.,Clinical and laboratory features of the Nocardia spp. based on current molecular taxonomy. Clin Microbiol Rev. 2006;19(2):259–82. 10.1128/CMR.19.2.259-282.2006 16614249 PMC1471991

[20] Gomez-Flores A, Welsh O, Said-Fernandez S, Lozano-Garza G, Tavarez-Alejandro RE, Vera-Cabrera L. In Vitro and In Vivo Activities of Antimicrobials against Nocardia brasiliensis. Antimicrob Agents Chemother. 2004;48(3):832–7. 10.1128/AAC.48.3.832-837.2004 14982772 PMC353153

[21] Bonifaz A, Flores P, Saul A, Carrasco-Gerard E, Ponce RM. Treatment of actinomycetoma due to Nocardia spp. with amoxicillin-clavulanate. Br J Dermatol. 2007;156(2):308–11. 10.1111/j.1365-2133.2006.07557.x 17223871

[22] Wortman PD. Treatment of a Nocardia brasiliensis mycetoma with sulfamethoxazole and trimethoprim, amikacin, and amoxicillin and clavulanate. Arch Dermatol. 1993;129(5):564–7. 8481016

[23] Poncio Mendes R, Negroni R, Bonifaz A, Pappagianis D. New aspects of some endemic mycoses. Med Mycol. 2000;38 Suppl 1:237–41. 11204151

[24] Fuentes A, Arenas R, Reyes M, Fernandez RF, Zacarias R. [Actinomycetoma and Nocardia sp. Report of five cases treated with imipenem or imipenem plus amikacin]. Gac Med Mex. 2006;142(3):247–52. 16875355

[25] Ameen M, Arenas R, Vasquez del Mercado E, Fernandez R, Torres E, Zacarias R. Efficacy of imipenem therapy for Nocardia actinomycetomas refractory to sulfonamides. J Am Acad Dermatol. 2010;62(2):239–46. 10.1016/j.jaad.2009.06.043 20005007

[26] Baril L, Boiron P, Manceron V, Ely SO, Jamet P, Favre E, et al Refractory craniofacial actinomycetoma due to Streptomyces somaliensis that required salvage therapy with amikacin and imipenem. Clin Infect Dis. 1999;29(2):460–1. 10.1086/520246 10476772

[27] Organization WH. Mycetoma: World Health Organization; 2019 [cited 2019 October 1]. Available from: https://www.who.int/news-room/fact-sheets/detail/mycetoma.

